# Restraint devices for repetitive transcranial magnetic stimulation in mice and rats

**DOI:** 10.1002/brb3.1305

**Published:** 2019-04-29

**Authors:** Chengliang Zhang, Rulan Lu, Linxiao Wang, Wenwei Yun, Xianju Zhou

**Affiliations:** ^1^ Laboratory of Neurological, Department of Neurology The affiliated Changzhou No.2 People’s Hospital of Nanjing Medical University Changzhou China; ^2^ Department of Neurology Integrated Hospital of Traditional Chinese Medicine, Southern Medical University Guangzhou China

**Keywords:** mouse, rat, repetitive transcranial magnetic stimulation, restraint device

## Abstract

**Introduction:**

Repetitive transcranial magnetic stimulation has been widely used for the treatment of neurological and psychiatric diseases. Rodent animals including mice and rats are often used to investigate the potential cellular and molecular mechanisms for the therapeutic effects of repetitive transcranial magnetic stimulation. So far there is no report about an easy‐to‐use device to restrain rodent animals for repetitive transcranial magnetic stimulation.

**Methods and Results:**

We introduced the design and use of the restraint device for mice or rats. In the mouse device, western blot and real‐time PCR analysis showed that，in stimulated mouse frontal cortex, 10 Hz high frequency stimulation for 10 sessions resulted in enhanced expression of NR2B‐containing N‐methyl‐D‐aspartic acid receptors and reduced α1 subunit of inhibitory GABA_A_ receptors, whereas 0.5 Hz low frequency stimulation for 10 sessions caused decreased expression of NR2B subunit and increased α1 subunit of GABA_A_ receptors. In the rat device, measures of motor evoke potentials indicated that 10 Hz stimulation for 10 sessions increased the excitability of stimulated cortex, whereas 0.5 Hz for 10 sessions reduced it.

**Conclusions:**

These results suggested the effectiveness of the devices. Thus, the two devices are practical and easy‐to‐use to investigate the mechanisms of repetitive transcranial magnetic stimulation.

## 1 BACKGROUND

Repetitive transcranial magnetic stimulation (rTMS) has been widely used for the treatment of neurological and psychiatric diseases, such as Alzheimer's disease, Parkinson's disease, epilepsy, stroke, depression and anxiety disorder, and sleep disorder (Cao et al., 2018; Feng, Zhang, Zhang, Wen, & Zhou, 2019; Lefaucheur et al., 2014; Zhang et al., 2019). However its therapeutic mechanisms are not completely clear. Previous studies have used rodent animals to investigate its potential cellular and molecular mechanisms related to synaptic plasticity (Guo, Lou, Han, Deng, & Huang, 2017; Ji et al., 2013; Tang, Thickbroom, & Rodger, 2015). Unlike humans, it is not practical to keep animals (including mouse or rat) stationary during the delivery of longer rTMS period. Therefore, it is necessary to take some measures to restrain the animals. In previous literature, investigators used the anesthesiology method (Sasso et al., 2016; Sykes et al., 2016), or by hand (Hesselberg, Wegener, & Buchholtz, 2016; Lim, Lee, Yoo, & Kwon, 2014; Sasso et al., 2016), or cloth, bag and straps (Ljubisavljevic et al., 2015); Tang et al., 2018), or some undefined devices (Guo et al., 2014). In animals, rTMS is often used 1–2 times each day (for minutes to tens of minutes each time), lasts for a few of days or tens of days (Fleischmann & Hirschmann, 1999; Guo et al., 2017; Ji et al., 2013). Thus, daily anesthesia may have potentially adverse effects in animals; Restraint by hand or by cloth, bag and tape may be laborious, or be potentially inconvenient for the delivery of rTMS, especially for a longer stimulation time (over 10 min). Here we designed the restraint device for mice and rats, respectively. The two devices would be practical and easy‐to‐use in exploring the therapeutic mechanisms of rTMS.

## 2 MATERIALS AND METHODS

### 2.1 Animals

Animals were purchased from Changzhou Cavens Lab Animal Co. Ltd. Adult male C57/B mice (22–28 g) and *SD* rats (220‐280g) were used. Animals were raised at 25°C and 60% humidity and in 12‐hr light: 12‐hr dark. Animal experiments were carried out according to Institutional Animal Care and Use committee (IACUC) of Nanjing Medical University and were approved by the ethics committee of the Affiliated Changzhou No.2 People's Hospital (No. 2014Keyan002‐01).

### 2.2 Anesthesia

Only Rats were intraperitoneally (i.p.) injected with sodium pentobarbital (Sigma, St. Louis, USA) at 70 mg/kg for anesthetization. After anesthetization, rats were placed on a brain stereotaxic frame (Stoelting Wood Dale, IL) as described previously (Muller et al., 2014), and the corresponding measures were taken to avoid the disturbance of potential electrical current during MEP measurement.

### 2.3 Electromyographic recording

Motor evoked potentials (MEPs) were recorded with 26G stainless steel needle electrodes which were inserted into the right brachioradialis muscle of rats as described elsewhere (Rotenberg et al., 2010). A reference electrode was inserted into the right paw and a ground electrode was inserted into the rat tail. The muscle was determined by touching the stretched forelimb. Electromyographic (EMG) signal was collected and processed by biological signal acquisition and processing system (MedLab‐U/4C501H, Nanjing Meiyi Tecnology Co., Nanjing, China). The EMG signal was amplified ×1,000 and band pass filtered 10‐1,000Hz, and digitized with 40 Hz sampling. Before rTMS (day 0) and 6 hr after rTMS (Day 5), MEPs were performed (*n* = 6 for each group).

### 2.4 Transcranial magnetic stimulation

A 70mm figure‐of‐eight coil (YIRUIDE, Wuhan, China, 3.0 Tesla) was placed on the left head scalp of the rat. The center of the coil was placed over the left motor cortex as described previously (Muller et al., 2014). Pulses were delivered at 1Hz to obtain optimal stimulation site where the maximal amplitude of MEPs were elicited by adjusting the coil location. By visual inspection, such lateralized TMS induced a twitch only in the right forepaw and shoulder. Resting motor threshold (rMT) was defined as the minimal stimulation intensity to obtain MEPs of more than 20 µV peak‐to‐peak amplitude in at least five of 10 trials. For low frequency rTMS group, the parameters were as follows: 0.5 Hz, 600 pulses, 20 min, 20% intensity of machine output for mice (causing no significant muscle twitch in the extremities) and 80% rMT for rats, in consecutive five days (one session in the morning and one session in the afternoon). For high frequency rTMS group, 10 Hz, 1 s stimulation, 9 s interval, 10 min stimulation, a total of 600 pulses, 30% intensity of machine output for mice (causing significant muscle twitch in the extremities) and 110% rMT for rats, in consecutive 5 days (one session in the morning and one session in the afternoon). Sham rTMS was delivered by the change of the coil positioning (perpendicular to the head scalp) with a same protocol of high frequency rTMS. To reduce the stressful responses, the naive animals were put into the restraint device three times (lasting for 1–2 min each time) one day before the whole rTMS protocol in order to adapt to this restraint condition.

### 2.5 Western blot and real‐time PCR

Before rTMS (day 0) and 6 hr after rTMS (Day 5), the two different groups (*n* = 3 for each group) were separately sacrificed by cervical dislocation and the brains were quickly removed. Then the front cortex was separated, cut two parts (one for western blot and the other for real‐time PCR), and stored at 80℃ until further use.

Western blot was carried out based on our previous study (Chen et al., 2016). The target proteins were immunoblotted with primary antibody overnight at 4^◦^C to anti‐NR2B (1:1,000, Cell signaling Technology) and anti‐GABA_α1_R (1:10,000, Millipore) and anti‐β‐actin (1:10,000, Sigma), followed by incubation with horseradish peroxidase‐conjugated goat anti‐rabbit secondary antibody (1:10,000; Cell Signaling Technology). The blots were then thoroughly washed and subjected to ECL detection (SuperSignal West Pico; Pierce, Rockford, IL). The signal intensity was determined using ScionImage software (Scion Corp., Frederick, MD). The NR2B subunit and GABA_α1_R protein level was normalized to β‐actin.

Real‐time PCR was performed by using SYBR® Select Master Mix (Applied Biosystems, Austin, TX) in a ViiA™ 7 real‐time PCR system. Total RNA from cortical tissue (*n* = 3 for each group) before stimulation and after stimulation was extracted using the NucleoSpin RNA Kit (MN, Düren, Germany) based on the instructions of the manufacturer. RNA concentration was measured using NanoDrop 1,000 (Thermo, Waltham, MA). Total RNA (1 µg) was reverse transcribed to cDNA with the High Capacity cDNA Reverse Transcription kit (Applied Biosystems, Foster City, CA). Primer sequences used were as follows: NR2B, 5′‐AACCTCCTGTGTGAGAGGAAA‐3′ (forward) and 5′‐CGGGGATAGAAAGGCAGCTT‐3′; GABAα1R, 5′‐ACCATGCCTAATAAGCTCCTGCGT‐3′ (forward) and 5′‐CAAGTGCATTGGGCATTCAGCTCT‐3′ (reverse); β‐actin (as an internal control), 5′‐TCTTGGGTATGGAATCCTGTGGCA‐3′ (forward and 5′‐ACAGCACTGTGTTGGCATAGAGGT‐3′ (reverse). The comparative threshold cycle method was utilized for relative mRNA quantification. All values were normalized to the control and expressed as fold change relative to control.

### 2.6 Statistical analysis

Statistical analysis was performed using Prism 6 (GraphPad Software, San Diego, CA). High frequency rTMS and low frequency rTMS were regarded as two independent and significantly different treatments, thus student's test was used. Values in real stimulation group were normalized to that in sham stimulation, and then comparisons were made between pre‐rTMS and post‐rTMS. *p* < 0.05 was considered significant.

### 2.7 Design of the restraint devices

**Figure 1:**
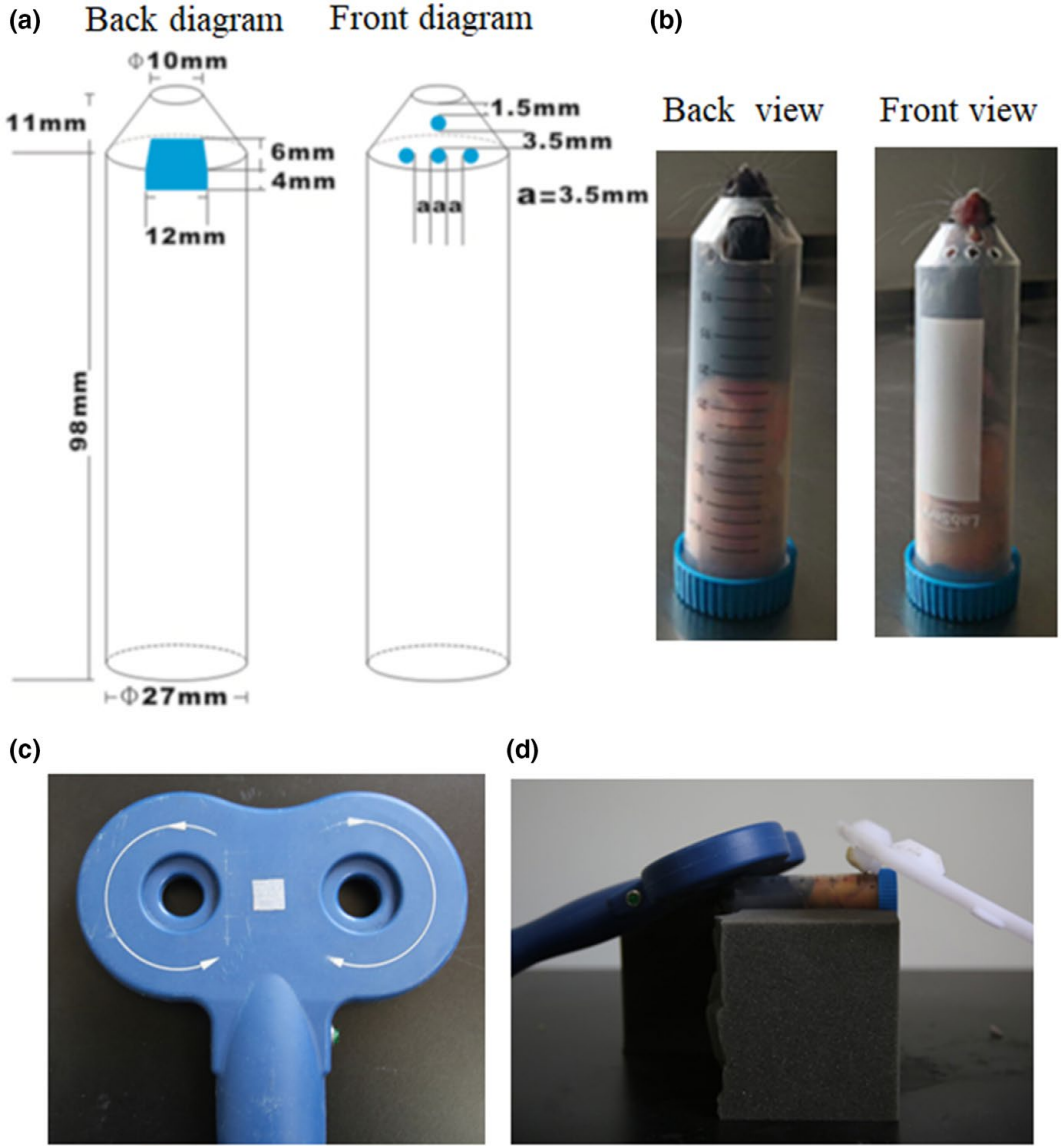
Mouse restraint device for rTMS. (a) A schematic diagram (back and front) of the mouse device; (b) A practicality picture (back and front view) of the mouse device; (c) A medical adhesive tape was attached in the center of the eight‐shaped coil; (d) The mouse device was placed on the sponge platform for rTMS

**Figure 2:**
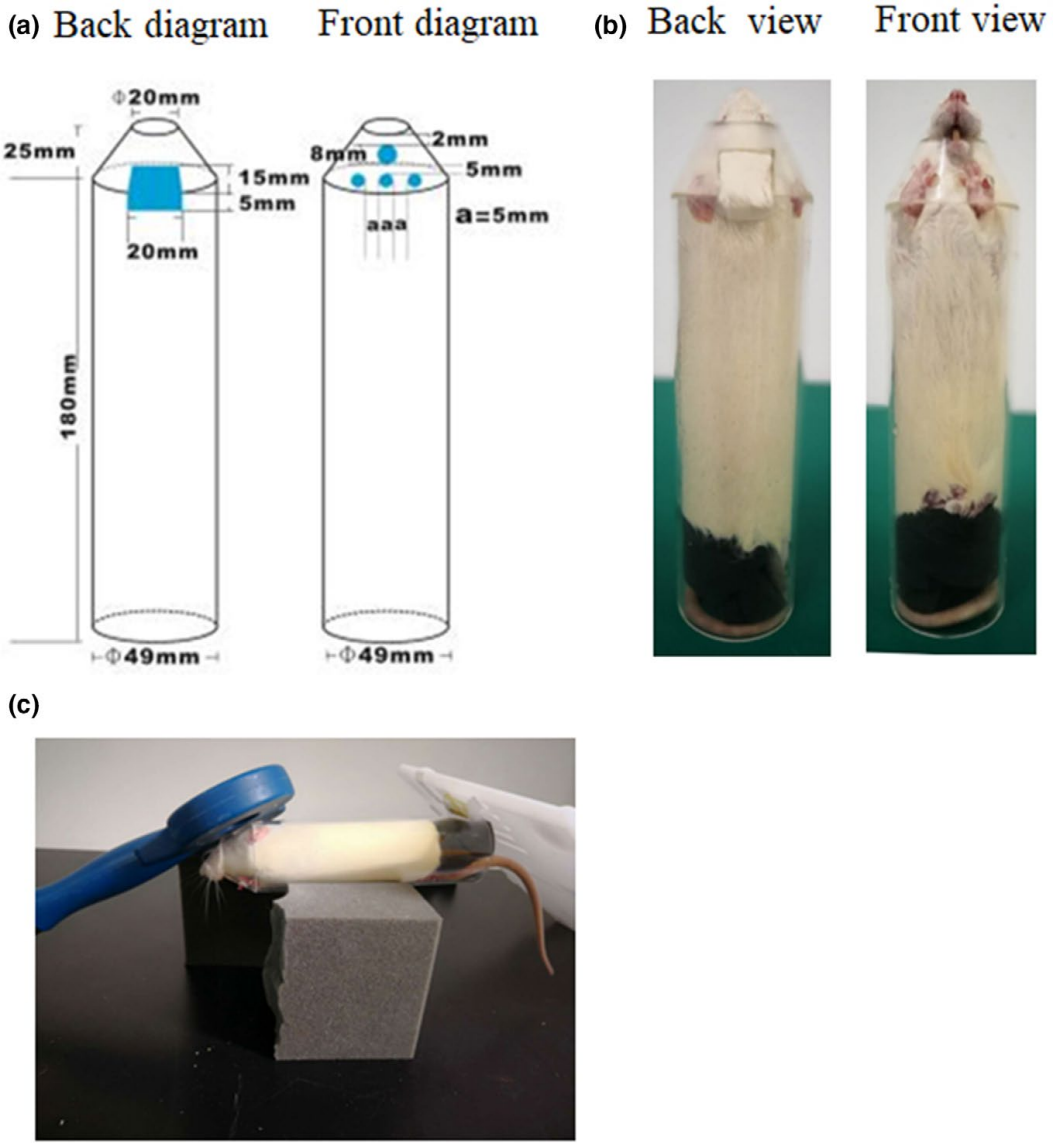
Rat restraint device for rTMS. (a) A schematic diagram (back and front) of the rat device; (b) A practicality picture (back and front view) of the rat device; (c) The rat device was placed on the sponge platform for rTMS

Based on previous studies, we found that the 50 ml‐centrifuge Corning tube commonly used in laboratory was suitable for the restraint of adult mice (Koh, Ji, Kim, Lee, & Kim, 2015; Zhang et al., 2008). The conical part at the bottom was suitable for accommodating the head of the mouse, while the circular tube was suitable for accommodating the body part of the mouse. The key was to expose the mouth of the mouse for breathing and to place the teeth into a hole according to the design principle of the mouse brain stereotaxic apparatus. In addition, the site for rTMS on the head needed to be exposed when the whole body was restrained. Actually, the change in the head size of adult male weighing 22 g to 28 g was relatively small. The difference in body size in the tube could be adjusted using a flexible sponge at the restraint. First, we used a 50‐ml centrifuge tube, and made a round outlet with inner diameter of 1 cm (where the rostral part of the mouse was exposed from the tube for breathing) at the conical end of the tube (Figure 1a,b). Then a rectangular window with a size of 1.2 cm length × 1.0 cm width was dug out at the junction of conical tube and circular tube (Figure 1a,b). One side of the window on the circular tube was just located on the lowest scale line on the outer surface of the circular tube. On the outer surface of the tube, the file was used to file the small window into a plane on which the rTMS coil was placed. The window was used for exposure of the top of the mice head and matched the center of the coil of rTMS apparatus by the guidance of a medical adhesive tape （1.2 cm × 1.0 cm）attached to the center of the 8‐shaped coil (Figure 1c). On the opposite side of the window, four holes with a diameter of 3.5 mm were drilled out. One of them was used to hold mandibular teeth of mice (Figure 1a,b), which was 1–1.5 mm from the round exit. The other three holes were used for ventilation and forelimb support, which were just below the first hole. The mouse device was for mice with 22–28 g weight. Beyond the weight range, it may be necessary to modify these sizes. But the basic principle of design remains unchanged. For the rat device, based on the same principle of design and the size of head and body of adult rats, we made a new device with different sizes using 2 mm wall thickness plexiglass material after several trials (Figure 2a,b). Similarly, the rat device was for rats with a range of 220–280g weight. Also, it will be also necessary to modify these sizes beyond the range.

### 2.8 The use, verification and discussion for restraint devices

When using the two devices, the experimenter guided the animals into the devices with their hands according to the mouse favorite habit of entering holes, and then covered the window on the tube with their left hand and pushed the mouse forward with their right hand until the rostral part of the animals protruded from the round outlet. Quickly, a piece of elastic sponge with a suitable size was inserted into the devices against the caudal part of the mouse, and then the head position was adjusted until the mandibular teeth entered the first hole and the top of the head was exposed to the window (Figures 1b and 2b). Next, the devices were placed on a sponge platform. The center of the 8‐shaped coil was placed over the small window, tangentially contacting the scalp of animals. At the other end of the tube, a plastic plate was used for balance (Figures 1d and 2c). Due to the smaller brain size and the larger coil, it would be difficult to locate precisely the stimulation site in animals. If researchers use other coils (such as loop coils) or custom animal coils, a small window can be made at the top of the head according to the shape of the coils. If researchers roughly locate the stimulation site at one side of the animal brain, a small window might be opened in the corresponding part of the devices.

**Figure 3:**
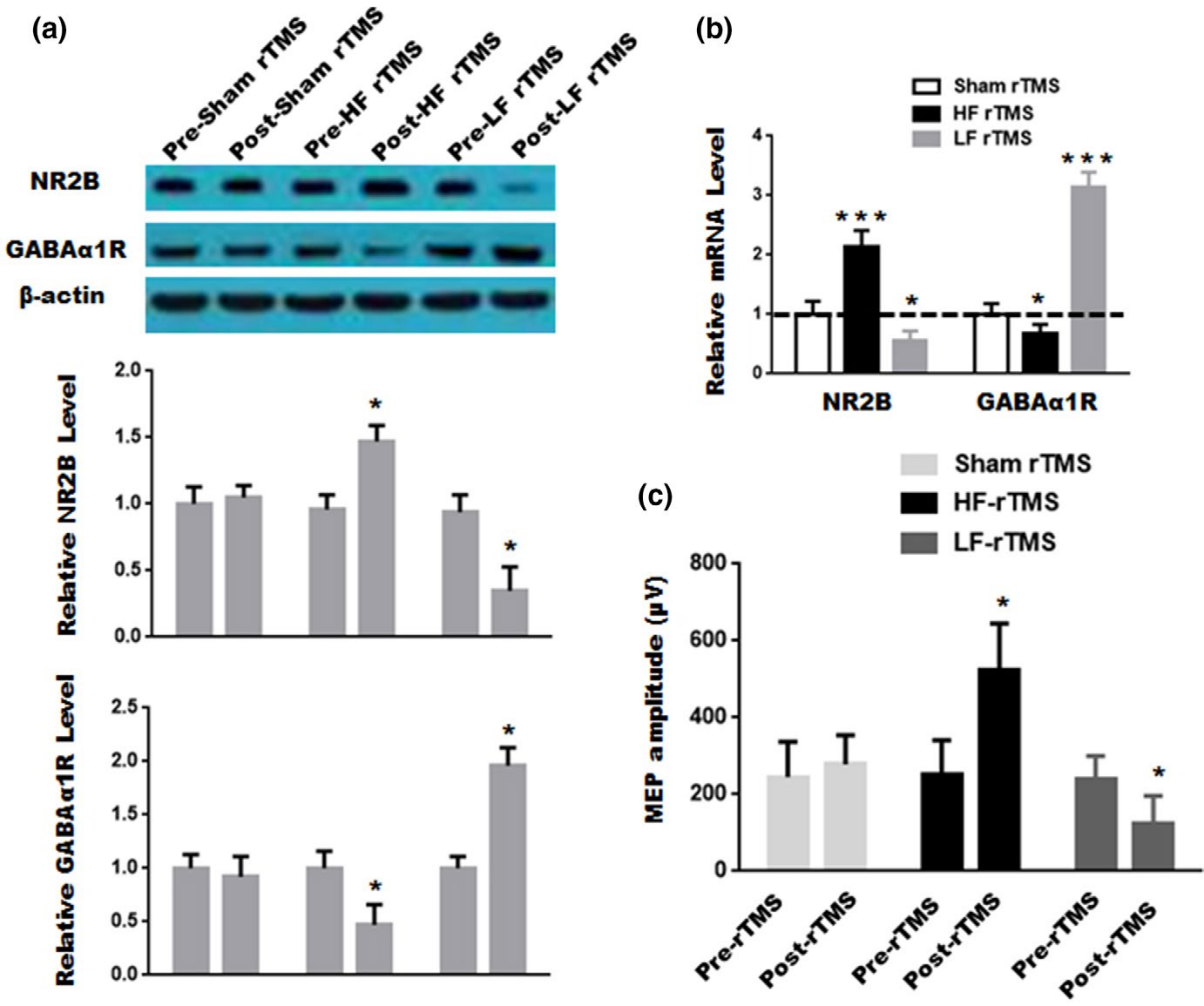
Verification of effectiveness of restraint devices. (a) The proteins were analyzed from the mouse frontal cortex in the sham rTMS group, the high frequency rTMS group and the low frequency rTMS group before stimulation (pre‐rTMS) and after stimulation (post‐rTMS). Data are presented as mean ± *SD* (*n* = 3 for each group). Upper panel, representative blots; Middle and Lower panel, quantitative analysis for NR2B subunit and GABA_α1_R expression, respectively.; *p < 0.05, compared to pre‐rTMS. (b) The expression of NR2B mRNA, GABAα1R mRNA and β‐actin mRNA were analyzed from the sham rTMS group, the high frequency rTMS group and the low frequency rTMS group. Data are presented as mean ± *SD* (*n* = 3 for each group). *p < 0.05, ***p < 0.001, compared to pre‐rTMS. (c) MEPs were detected before stimulation and after stimulation in anesthetized rats from the sham rTMS group, the high frequency rTMS group and the low frequency rTMS group. Data are presented as mean ± *SD* (*n* = 6 for each group). *p < 0.05, compared to pre‐rTMS. Values in real stimulation group were normalized to that in sham stimulation, and student's *t* test was used

To validate the effectiveness of the devices, high frequency (10 Hz) or lowfrequency (0.5 Hz) rTMS was delivered over the cortex of mice for 10 sessions, since previous studies showed that low frequency rTMS decreases cortical excitability, whereas high frequency rTMS increases cortical excitability in humans or animals (Maeda, Keenan, Tormos, Topka, & Pascualleone, 2000; Nielsen & Jacobsen, 1997; Muller et al., 2014). We examined the change in the NR2B subunit‐containing N‐methyl‐D‐aspartic acid (NMDA) receptor (a key subunit of excitatory glutamate receptors in determining synaptic changes) (Rudolph & Möhler, 2006; Zhou, Ding, Chen, Yun, & Wang, 2013) and the α1 subunit of inhibitory γ‐aminobutyric acid (GABA) A type receptor (GABAα1R, a most prominent subtype)(Rudolph & Möhler, 2006; Zhou et al., 2013) in mice using western blot and real‐time PCR. As illustrated in Figure 3a, as compared to pre‐rTMS, low frequency rTMS of 10 sessions reduced significantly the expression of NR2B subunit in the frontal cortex of mice but increased the expression of GABA_α1_R. In contrast, high frequency rTMS of 10 sessions significantly enhanced the expression of NR2B subunit in the mouse frontal cortex, but decreased GABA_α1_R expression. Similar results were obtained in the expression of NR2B mRNA and GABA_α1_R mRNA of the frontal cortex by low frequency or high frequency rTMS (Figure 3b). To further recapitulate previous results, we examined resultant cortical excitability induced by 0.5Hz rTMS or 10Hz rTMS for 10 sessions by measuring MEP amplitude reflecting cortical excitability in rTMS‐stimulated cortex of rats. As expected, after 10 sessions of 0.5Hz rTMS, the MEP amplitude was significantly reduced as compared to pre‐rTMS (Figure 3c). On the contrary, 10 Hz rTMS led to a marked increase in MEP amplitude (Figure 3c). There was no obvious difference in MEP amplitude between pretreatment and posttreatment in sham rTMS group. Taken together, these results suggested that the two devices are feasible.

As mentioned above, previous studies stimulated the cortex of animals to investigate molecular and cellular mechanisms for the therapeutic effects of rTMS with different restraint methods. For a short stimulation period (3–5 min), restraining animals by hand during the delivery of rTMS appeared not to be a problem. However, when restraining animals for a longer period (over 10 min) by hand, it would be laborious. Additionally, slightly restraining animals using a restraint bag seemed not to be laborious (Tang et al., 2018), but it may be potentially inconvenient for the delivery of rTMS. Daily anesthesia for over 10 days may have potentially adverse effects in animals. To solve these problems, we designed the restraint devices to improve the delivery of rTMS and replicated some previous conclusions in humans or animals (Maeda et al., 2000; Muller et al., 2014) to confirm the effectiveness. As compared to previous restraint methods, the methods appeared to more practical, especially making the delivery of rTMS more convenient (Hesselberg et al., 2016; Lim et al., 2014; Ljubisavljevic et al., 2015; Sasso et al., 2016). However, their usefulness and effectiveness need to be verified by more researchers in the future.

The disadvantages of the devices were to squeeze lightly the chest of animals, leading to stressful responses. Therefore, the sponge was not to be tucked too tightly. To minimize the stressful responses, the naive animals would be put into the device three times (lasting for 1–2 min each time) one day before the whole experiment in order to adapt to this condition. After adaptation, animals did not show any significant struggle. Additionally, the rat device may be conveniently used for blood collection or injection into the tail vein.

## CONFLICT OF INTERESTS

The authors declared no competing interests and signed.

## DATA AVAILABILITY STATEMENT

The data that support the findings of this study are available from the corresponding author upon reasonable request.
